# Chagas Disease in Pregnant Women in the Peruvian Amazon Basin. Cross-Sectional Study

**DOI:** 10.3389/fvets.2020.00556

**Published:** 2020-09-15

**Authors:** José-Manuel Ramos-Rincón, Sonia Ortiz-Martínez, María-Esteyner Vásquez-Chasnamote, Olga-Nohelia Gamboa-Paredes, Viviana-Vanessa Pinedo-Cancino, Cesar Ramal-Asayag, Miguel Górgolas-Hernández-Mora, Martin Casapía-Morales

**Affiliations:** ^1^Clinical Medicine Department, University Miguel Hernández de Elche, Alicante, Spain; ^2^Internal Medicine Service, General University Hospital of Alicante-ISABIAL, Alicante, Spain; ^3^Medical Practice El Ballestero, Health Service of Castilla La Mancha, Albacete, Spain; ^4^Natural Resources Research Center, Peruvian Amazon National University, Iquitos, Peru; ^5^Research Assistant, Amazon Rainforest Civil Association, Iquitos, Peru; ^6^Molecular Biology and Immunology Laboratory of the Specialized Unit of LIPNAA-CIRNA, Peruvian Amazon National University, Iquitos, Peru; ^7^Infectious Diseases and Tropical Medicine Service, Loreto Regional Hospital, Iquitos, Peru; ^8^School of Medicine, National University of the Peruvian Amazon, Iquitos, Peru; ^9^Infectious Disease Division, University Hospital Foundation Jiménez Díaz, Madrid, Spain; ^10^Medicine Department, Autonomous University of Madrid, Madrid, Spain; ^11^Medical Department, Amazon Rainforest Civil Association, Iquitos, Peru

**Keywords:** Chagas disease, *Trypanosoma cruzi*, pregnant, Peru, Amazon

## Abstract

**Aims:** To assess the prevalence of Chagas disease in pregnant women in Iquitos City, Peru.

**Material and Methods:** Cross-sectional survey in 300 pregnant women in Iquitos (Peru) from 1 May 2019 to 15 June 2019. Women were tested using an ELISA serology test.

**Results:** Serology was positive in one case (prevalence: 0.33%; 95% confidence interval: 7.1–13.9%), of a 25-year-old woman who lived in a wooden house with a leaf roof in a periurban area of Iquitos. She was familiar with kissing bugs and had chronic, asymptomatic Chagas disease.

**Conclusion:** The prevalence of Chagas disease is low in the urban and peri-urban area of the city of Iquitos.

## Introduction

Chagas disease is a systemic chronic parasitic infection caused by *Trypanosoma cruzi* that affects 6–7 million people in Central and South America. It is considered a neglected tropical disease and has a high public health impact in the area.

While mainly vector-borne, Chagas disease can also be spread via blood transfusion, transplantation, or from mother to child (1). The latter, congenital route is frequent and especially relevant in areas where there is no vector transmission by insects or blood transfusion. To accelerate the elimination of this transmission route, strategies and methods should be applied to screen, diagnose, and treat all infected pregnant women as well as their newborns and, where appropriate, other children as soon as possible (2).

Chagas disease is endemic throughout the Pacific southwest of Peru, known as the Greater Southern Region, in the departments of Arequipa, Moquegua, Tacna, Ayacucho and Apurimac. In the past decade, widespread infestation with the vector *Triatoma infestans* and active transmission of Chagas disease to humans have been documented in this area (3, 4). In addition, the northeastern departments of Cajamarca, Amazonas, San Martín, and Ucayali (400–1,000 m above sea level) have also detected the thriving presence of the *Panstrongylus herreri* vector (3). However, there is little knowledge about the prevalence and epidemiology of *Trypanosoma cruzi* in northern Peru (5); the most significant resources have been allocated toward research and control efforts in the south (5).

Iquitos is a city in the Peruvian Amazon Basin, in the Department of Loreto and near the confluence of the Marañon and Ucayali rivers. A few isolated cases of Chagas disease have been documented in the town and surroundings areas (6), but no survey has ever focused on the pregnant population. As these women can unknowingly transmit the disease to their newborns, its detection is highly recommended (2, 7). The aim of this study was to estimate the prevalence of Chagas disease in pregnant women in Iquitos, Peru, in the Peruvian Amazon basin.

## Methods

### Setting and Study Period

**Figure 1:**
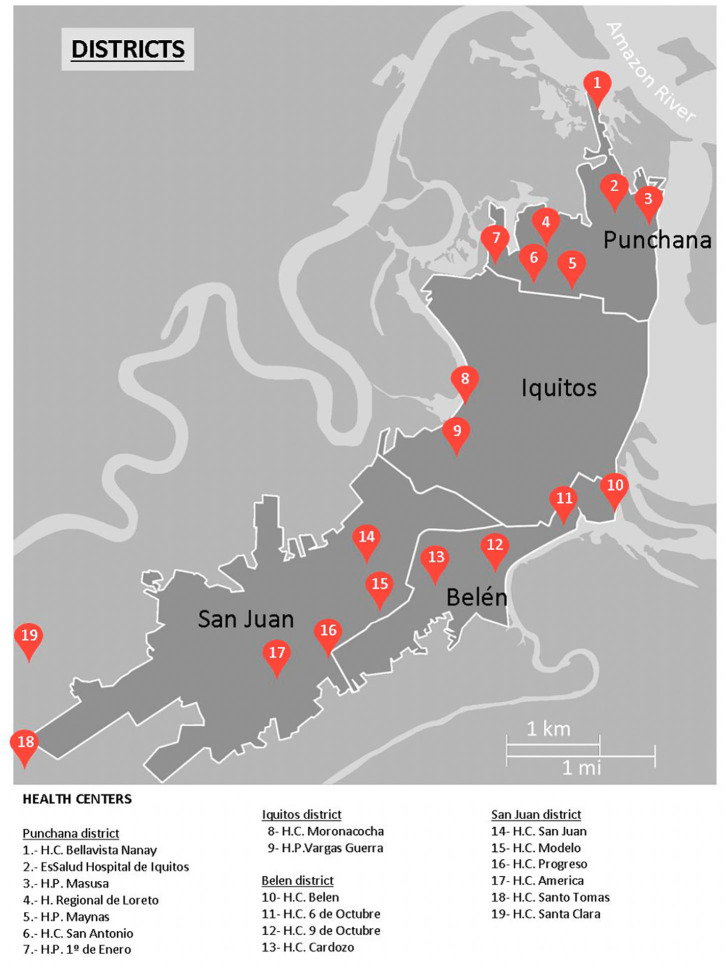
Health centers in Iquitos city and surrounding area.

We performed a cross-sectional survey of Chagas disease, strongyloidiasis, and human T-cell leukemia-lymphoma virus (HTLV) infection in an urban and periurban area of Iquitos (Peruvian Amazon) (Figure 1), from 1 May 2019 to 15 June 2019.

### Study Population

We included pregnant women attending the health centers in four districts of the greater Iquitos area, located in the Peruvian Amazon. Participants were adults over the age of 18 years and were selected through convenience sampling (i.e., on days when the researcher was at the health center) when they visited the midwife during prenatal check-ups. All participants were informed about the screening and signed informed consent.

### Data Collection

On enrollment, participants were asked about sociodemographic variables and their familiarity with Chagas disease. We obtained a blood sample for serological detection of *T. cruzi* antibodies, *S. stercoralis* IgG antibodies, and HTLV antibodies, and a stool sample to test for parasitic infections. Our group published results on the prevalence of strongyloidiasis and other intestinal parasites in a parallel report (8).

Detection of *T. cruzi* IgG antibodies was performed by two different assays: the Chagatest ELISA lysate (Wiener, Rosario, Argentina) and the Chagatest ELISA recombinant v.4.0 (Wiener, Rosario, Argentina). *T. cruzi* infection was considered confirmed if both tests yielded a positive result, while participants were considered seronegative when both tests yielded negative results. Specimens with only one positive test were considered inconclusive. The Chagatests were completed according to manufacturer's instructions and the threshold for positive results was 0.10 optical density (OD) units above the mean absorbance of two negative control specimens included on each plate.

### Data Analysis

The collected data were systematically recorded and analyzed using IBM SPSS Statistics, version 22.0. We used the chi-squared test or Fisher's exact test to determine the presence of Chagas disease according to several demographic variables, considering results to be significant where the *P* < 0.05.

## Results

The study included 300 pregnant women with a mean age of 26.7 years (standard deviation [SD] 6.4; range 13–38), mean of number of deliveries of 2.9 (SD: 1.7), and a mean gestation period of 172 days (SD: 59). Just under half (44.7%) lived in peri-urban areas, while the rest lived in the city. Table 1 shows the sociodemographic and epidemiological characteristics of the participants in the study. Figure 1 Health centers in Iquitos city and surrounding area.

**Table 1:** Epidemiological characteristics of pregnant women.

**Variables**	**Frequency**
**Sociodemographic conditions**	
Mean age (years)	26 (SD: 6.4)
Mean time living in the place (years)	9.9 (SD: 9.7)
Residence in rural area	134 (44.7%)
**Characteristics of pregnancy**	
Mean gestational age (days)	172 (SD: 59)
Primiparous	64 (21.3%)
Mean number of pregnancies	2.9 (SD: 1.7)
Mean number of children alive	1.8 (SD: 1.5)
**Risk factors for Chagas disease**	
History of blood transfusion	15 (5.0%)
**Knowledge of Chagas disease**	
Any knowledge about Chagas disease	10 (3.3%)
Contact with person with Chagas disease	0 (0.0%)
**Characteristics of living houses**	
Wood house	286 (95.3%)
Leaf roof	234 (78.0%)
Soil floor	89 (29.7%)

*SD, standard deviation*.

Four participants tested positive on the Chagatest ELISA recombinant v.4.0 with titers > 0.2, but only one had a second positive serology test (Chagatest ELISA lysate) (Table 2). Therefore, just one participant (0.33%, 95% CI: 0.02–2.13%) was considered as a definitive positive for Chagas disease. Three other participants had inconclusive results by both ELISAs. Their infection status therefore remained unresolved, and their data were excluded from further analysis. There were no statistically significant differences in the sociodemographic conditions, knowledge of Chagas disease, or housing conditions in cases with positive and negative serology against *T. cruzi*.

**Table 2:** Results of two serological procedures in positive cases.

**Code**	**Age/health center**	**Antecedents blood transfusion**	**Chagatest ELISA recombinant v.4.0 titers**	**Chagatest ELISA lysate titers**	**Chagas disease**
1	25 years/santa clara	No	1.118	1.416	Yes
2	19 years/san juan	No	0.267	0.011	Inconclusive
3	33 years/san juan	No	0.237	0.029	Inconclusive
4	19 years/San juan	No	0.301	0.038	Inconclusive

The positive case was a 25-year-old woman who lived in a periurban area of Iquitos (Figure 1). She had been living in the same house—a wooden construction with a leaf roof—for the last 5 years. She had two other children and was familiar with the “chirimacha” (kissing bugs or triatomines) and reported seeing them at home, but she did not remember being bitten by one. She had not received any blood transfusion and did not have any symptoms of Chagas disease (chest pain, palpitation, dysphagia). Her electrocardiogram was normal, showing a repolarization disorder.

## Discussion

Our study demonstrates that the prevalence of asymptomatic Chagas disease in pregnant women is low (0.33%) in the Iquitos area of the Peruvian Amazon Basin. This evidence helps to fill gaps in knowledge arising from the few seroprevalence studies in pregnant women in Peru (7). In 2005, a study performed in Arequipa (southern Peru) in 3,000 pregnant women showed serological positives in 22 (0.7%) participants; only one newborn was IgM positive (9).

The prevalence of Chagas disease in pregnant women, both in our study and the one performed in Arequipa, is low compared to those performed in Bolivia, northern Argentina, or Brazil, which have reported a prevalence of 21, 12.1, and 1.1%, respectively (10–12). In the general population, the literature reports a seroprevalence of Chagas disease in the southern department of Arequipa ranging from 2 to 5.8% (4, 13, 14). In the north (La Joya and Cajamarca departments), prevalence is slightly higher, between 7.6 and 14.9% (5, 15). Our results from Iquitos are sensibly lower than in these reports from elsewhere in Peru.

However, our results are relevant because they show that Chagas disease, although uncommon, is actually present in the Iquitos area. Asymptomatic Chagas disease cases like the one detected in our survey do occur. Other acute cases have previously been reported in 2006 and 2008 in the Amazon area (6, 16, 17). Therefore, Chagas disease is present not only in the jungle of northern Peru (5) but also in the Amazon basin. That said, seroprevalence of *T. cruzi* in pregnant women appears to be lower than that reported in the endemic areas of Peru (9). There may be small pockets of vector transmission of Chagas disease in Iquitos. Performing small studies around this case could help to uncover more; this adaptive strategy could efficiently identify secondary cases (13).

Chagas disease has been studied elsewhere in the Amazon basin, including in Ecuador [prevalence 3.6% (18)] and in Brazil (seroprevalence 1.5%). Throughout all these areas, Chagas continues to pose a threat to public health, one amplified by deforestation and its associated changes in transmission vectors (19, 20). Indeed, this region is at risk of transmission due to triatomine-contaminated food (21). Several initiatives, like the Iniciativa Andina (IA), have been launched in priority areas of Latin America to ensure the interruption of vector-borne transmission of Chagas as well as to improve surveillance and disease prevention (22).

The presence of pregnant women in the Amazon Basin who are at risk of congenital transmission to the fetus, along with other reported cases of acute Chagas Diseases (6, 16, 17) should serve as a warning of an (emerging) problem in this area of Peru. While the low prevalence does not justify screening in pregnant or puerperal women, other measures are called for. WHO/PAHO recommends interventions to disrupt vector transmission, including the elimination of the triatomines or other vectors from the study area; another possibility is assessing seroprevalence of *T. cruzi* infection in children 5 or younger (23). Several environmental and social factors may also directly or indirectly influence the biology and behavior of triatomides (24). Spatial clustering of infestation in the urban context may both challenge and inform surveillance and control of vector reemergence after insecticide intervention. These measures have been performed in several departments in Peru, such as Moquegua and Tacna, which were subsequently declared free of vector transmission by the WHO/PAHO (25).

Entomologic investigation of *T. infentans* and other triatomides is important for knowing the ecology of vector transmission (26). It is important to implement measures for controlling transmission of *T. cruzi* by triatomines in the Amazon basin, with vector surveillance and control with insecticide as has been happening in other parts of Peru (5). In light of our exploratory results and other cases reported in the area, it could be of interest to perform similar studies to those carried out in other parts of Peru (15, 17, 23). Another topic to investigate in the area is dogs, which are important reservoirs of *T. cruzi* and may play a role in reinitiating transmission in previously sprayed areas. Dogs may also serve as indicators of reemerging transmission (24).

Another potential line of research about Chagas disease in the Peruvian Amazon basin is the risk of oral transmission. In Brazil, Venezuela, Colombia, Bolivia, and French Guiana, several outbreaks of orally transmitted Chagas disease have been reported; these have been epidemiologically associated with the consumption of beverages like açai juice (the fruit from a species of palm tree) or sugar cane juice (27). Some cases of Chagas disease have also been described in young indigenous people who drank contaminated juice (17, 26). This line of research should continue in the area, as should health education, in order to prevent the contamination of juice with *T. infestans* excreta (26).

This study has some limitations. First, a complete study of congenital transmission was not performed. Secondly, there were three participants with borderline results according to both ELISAs. As this situation might reflect a window period, it would be necessary to re-test them after 6 months to confirm or rule out infection. Finally, there was no entomologic investigation about species living at the patient's home.

## Conclusions

Although the prevalence of Chagas disease is low in the urban and peri-urban area of the city of Iquitos, it is relevant to advise local authorities that the disease is actually present in the Peruvian Amazon Basin in pregnant women with a risk of congenital transmission. Our results indicate a probable low rate, but it is necessary to perform more studies and monitor the prevalence of the disease.

## Data Availability Statement

The raw data supporting the conclusions of this article will be made available by the authors, without undue reservation.

## Ethics Statement

The studies involving human participants were reviewed and approved by The Ethics Committee of the General University Hospital of Alicante (Spain) approved the project (PI2018/113), as did the Ethics Committee of Loreto Regional Hospital in Iquitos (Peru) (027-CIEI-HRL-2019). The patients/participants provided their written informed consent to participate in this study.

## Author Contributions

J-MR-R: conceptualization, formal analysis, methodology, project administration, supervision, writing—original draft, writing-review and editing, designed the study, analyzed clinical data, prepared, and reviewed the manuscript. SO-M: formal analysis, investigation, project administration, software, supervision, writing—original draft, and writing-review and editing. M-EV-C: methodology and writing-review and editing. O-NG-P: data citation, software, supervision, and writing-review and editing. V-VP-C: data curation, methodology, project administration, and writing-review and editing. CR-A: investigation and writing-review and editing. MG-H-M: conceptualization, writing—original draft, and writing-review and editing. MC-M: conceptualization, project administration, writing—original draft, and writing-review and editing manuscript. All authors contributed to the article and approved the submitted version.

## Conflict of Interest

The authors declare that the research was conducted in the absence of any commercial or financial relationships that could be construed as a potential conflict of interest.
